# Bisphenol A induces cell cycle arrest in primary and prostate cancer cells through EGFR/ERK/p53 signaling pathway activation

**DOI:** 10.18632/oncotarget.23360

**Published:** 2017-12-18

**Authors:** Antonio Bilancio, Paola Bontempo, Marzia Di Donato, Mariarosaria Conte, Pia Giovannelli, Lucia Altucci, Antimo Migliaccio, Gabriella Castoria

**Affiliations:** ^1^ Department of Biochemistry, Biophysics and General Pathology, University of Campania “L. Vanvitelli”, Naples, Italy; ^2^ IRCCS, SDN, Naples, Italy

**Keywords:** BPA, prostate cancer, cell cycle, AR, erk

## Abstract

Bisphenol A (BPA) belongs to the class of chemicals known as endocrine disruptors and has been also involved in the pathogenesis and progression of endocrine related cancer such as breast and prostate cancers. Here, we have investigated the effect of BPA in human prostate cancer LNCaP cells and in human non-transformed epithelial prostate EPN cells. Our data showed that BPA induces the down regulation of cyclin D1 expression and the upregulation of the cell cycle inhibitors p21 and p27, leading to cell cycle arrest. Interestingly, we found that the BPA anti-proliferative response depends on a strong and rapid activation of epidermal growth factor receptor (EGFR), which stimulates ERK-dependent pathway. This, in turn, induces expression of p53 and its phosphorylation on residue Ser15, which is responsible for cell cycle arrest. EGFR activation occurs upon a cross talk with androgen (AR) and estradiol receptor-β (ERβ) which are known to bind BPA.

Altogether, these findings show a novel signaling pathway in which EGFR activation plays a key role on BPA-induced cell cycle inhibition through a pathway involving AR and ERβ/EGFR complexes, ERK and p53. Our results provide new insights for understanding the molecular mechanisms in human prostate cancer. On the other, they could allow the development of new compounds that may be used to overcome human prostate cancer resistance to endocrine therapy in promising target therapeutic approaches.

## INTRODUCTION

Bisphenol A (BPA; 4, 40-dihydroxy-2, 2 diphenylpropane; CAS 80-05-7) is an organic compound well known by chemists and biologists since the end of 19^th^ century. Due to its structure, it was initially hypothesized that it was endowed with an estrogenic activity. Nevertheless, only recently BPA has been reported to have hormonal effects in reproductive organs of female rat [1]. BPA has attracted great interest in the chemical industry as it is still currently used as a monomer in the production of plastic polymers, such as polycarbonate, and as a regulator of polyvinyl chloride polymerization. These materials are commonly used for the production of a huge amount of consumer products including, first of all, plastic bottles, feeding bottles, some medical devices, and many others. BPA can contaminate water and food through its releasing in the environment, where it can be considered as widespread environmental pollutant. In recent years increasing attention has been given to BPA since a very relevant amounts of BPA (even higher than 1mg/kg) have been detected in some foods, like vegetables, probably as consequence of leak from plastic irrigation devices [1–6].

However, the impact of BPA on human life and related negative-effects are linked to non-monotonic phenotypical effect on human tissues. Several findings report that exposure to BPA is generally associated with increased risk of cancer, in particular for so-called hormone-related cancers such as ovarian cancer, breast cancer and, although so far less investigated, prostate cancer. Sex steroids influence the development and progression of those mentioned cancers [7–12]; and it is generally accepted that the BPA effects in eukaryotic cells are mostly mediated by steroid receptors, including estrogen receptors (ER-α and -β), androgen receptor (AR), estrogen-related receptors (ERRs) and peroxisome proliferator-activated receptors (PPARs).

Accumulating evidence suggests that BPA affects prostate cells, thereby leading to proliferation of human prostatic adenocarcinoma LNCaP cells through activation of the endogenous androgen receptor (AR) mutant (AR-T877A) [13], and this has been suggested to favor transition of prostate tumors to castration-resistant prostate cancer (CRPC) with a unfavourable diagnosis and poor response to the current available therapies. However, BPA acts either on AR or on its mutated variants in a dose-dependent manner by eliciting different effects on prostate cancer (PCa) cells. In fact, treatment with low doses (e.g. 1 nM) of BPA stimulates the transcriptional activity of AR-T877A, and acts synergistically with androgen hormone at physiological concentrations (e.g. 1 nM). BPA binds to AR-T877A, displacing androgen hormone binding to its receptor in a non-competitive manner [14] and activates or potentiates the transcriptional activity of other functional AR mutated variants such as V715M, L701H and K580R (isolated from prostate tumor samples), and AR-T877S, AR-V715M and AR-H874Y (from human prostate carcinoma xenograft-derived 22Rv1 cells), whereas no effect was reported on wild-type AR [13]. In contrast, at high concentrations (e.g. 10 μM), it has been shown that BPA, although still affect AR transcriptional activity, seems to reduce proliferation of LAPC4 cells (expressing wild-type AR), LNCaP cells (expressing the AR-T877A mutant), and, to a lesser extent, androgen-independent 22Rv-1 cells (expressing the AR-H874Y mutant). BPA seems have no significant effect on proliferation of AR-negative/androgen-independent PCa cells, such as PC-3 or DU-145 [13, 15].

Nevertheless, the effects of BPA on prostate cancer development and progression are far from being fully elucidated and the mechanism of its action is unclear. In this report, we investigated the effect of BPA in human prostate cancer LNCaP cells and in human non-transformed epithelial prostate cells EPN on proliferation and the signaling pathway involved. LNCaP cells are endowed with AR-T877A mutant and βER isoform, and represent a suitable model for androgen dependent cell growth.

It is now definitely acknowledged that beside the classical model of the mechanism of action of steroid receptors that mainly relies on their property to activate transcription, the steroid receptors activate rapid non-genomic transduction pathways. Activation of rapid signaling is required for hormone induced cell growth. On the basis of this notion, we investigated in prostate cancer and in human non-transformed epithelial prostate cells whether the BPA interferes with the activation of such pathways and the possible activation is involved in the action of this compound on DNA synthesis. We found that 50 and 100 μM BPA induces cell cycle arrest in LNCaP and in human non-transformed epithelial prostate cells EPN through specific signal transduction activation.

## RESULTS

### BPA causes cell cycle arrest in human prostate cancer LNCaP cells

**Figure 1:**
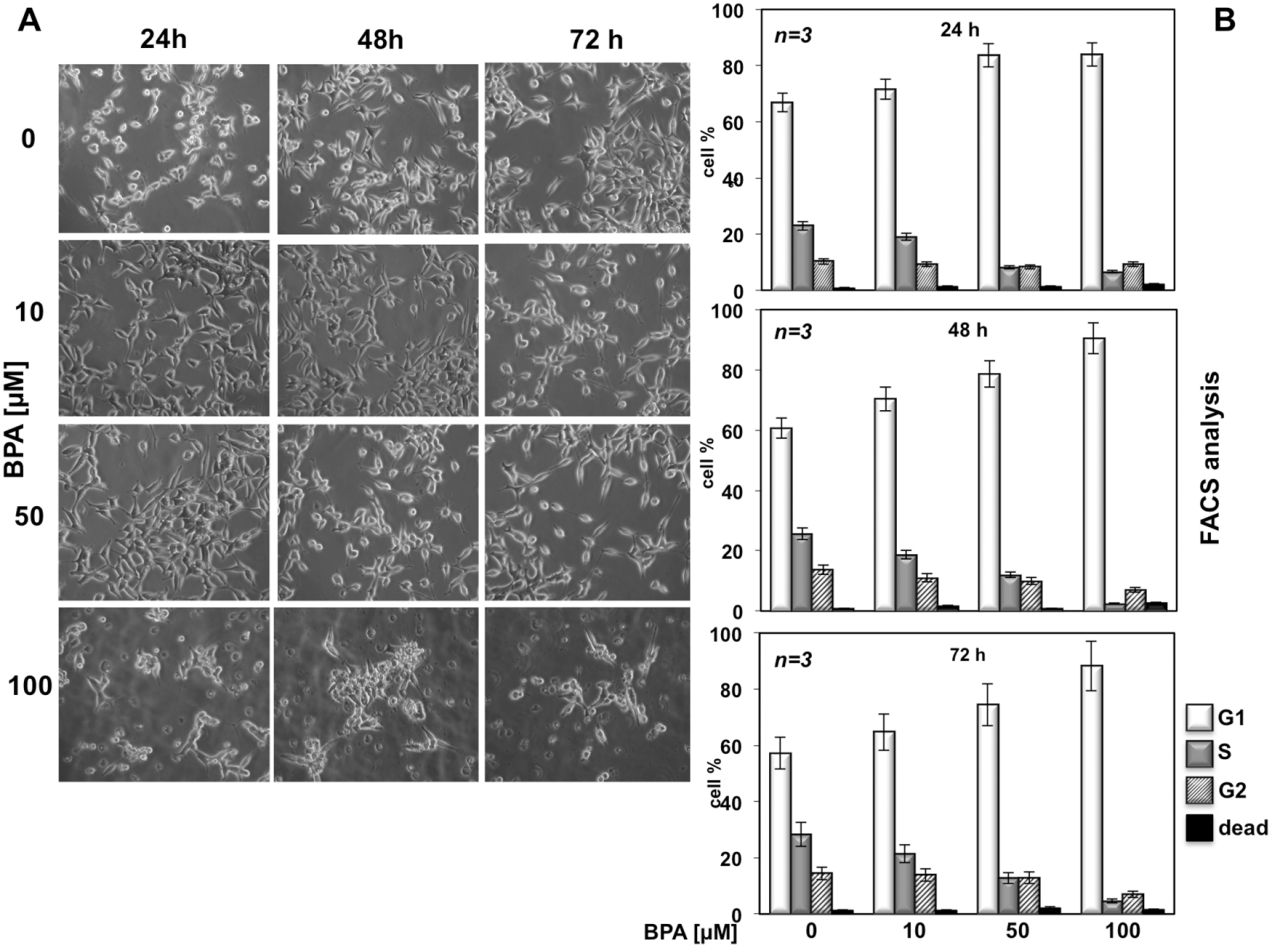
BPA affects LNCaP cell proliferation In **(A)**, LNCaP cells were plated at the same confluence in 100 mm dishes and unstimulated (0) or stimulated with 10, 50 or 100 μM Bisphenol A for 24, 48 and 72 hours. Images were captured with DMIRB inverted microscope (Leica) using N-Plan 10x objective (Leica) and a DFC 450C camera (Leica). They were analyzed with Application Suite (Leica) software and are representative of at least three independent experiments, each performed in duplicate. In **(B)**, cycling LNCaP cells were left unstimulated (0) or stimulated for the indicated times with Bisphenol A (BPA at 10, 50 and 100 μM). Cells were re-suspended and analyzed by FACS, as described in Methods. The graphs show means from three independent experiments and the distinct cell cycle phases as reported in Methods.

**Figure 2:**
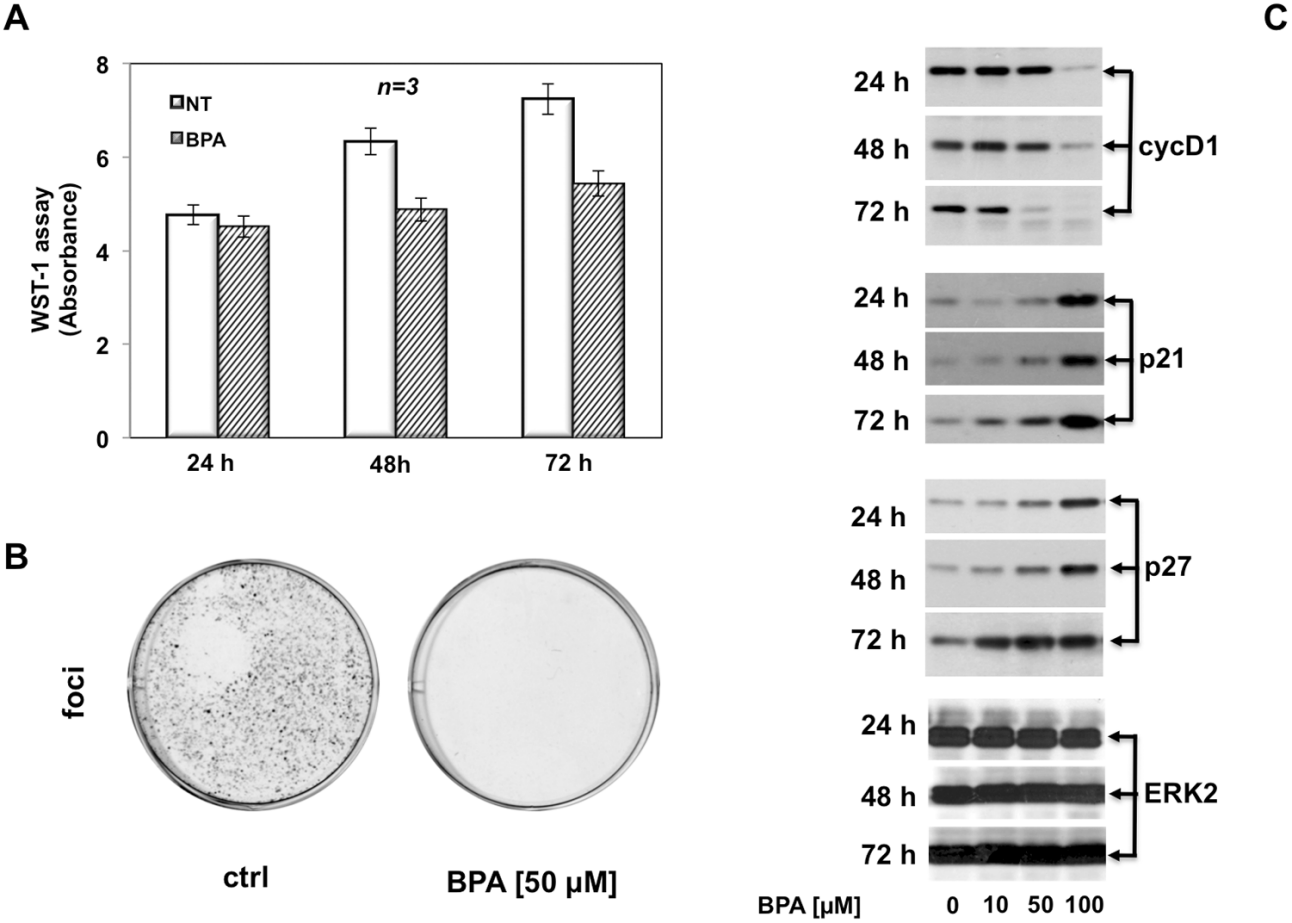
BPA halts cell cycle in LNCaP cells In **(A)**, cycling LNCaP cells, plated in 96-wells as described in Materials and Methods, were unstimulated or stimulated with 50 μM Bisphenol A for 24, 48 or 72 hours. Cell viability was assayed with WST-1 reagent. The graph in A represents the absorbance expressed as fold increase. In **(B)**, LNCaP cells were seed in 6 well plates and stimulated or unstimulated with 50 μM Bisphenol A. After 12 days the cells were fixed and stained as described in Materials and Methods. In **(C)**, LNCaP cells were untreated or treated with Bisphenol A (BPA at 10, 50 and 100 μM) for the indicated times and lysate proteins were analyzed by western blot, using antibodies against the indicated proteins (cyc D1: cyclin D1).

We previously reported that BPA induces MCF-7 cells proliferation [16] whereas BPA induces cell cycle arrest and apoptosis in three different acute leukemia cell line such as NB4, HL60 and K562 cells [17]. Based on this observation that BPA can have different effects depending on the cells used, we next evaluated the effects of BPA in LNCaP, a cancer cell line derived from human epithelial prostate cancer. We first evaluated whether BPA treatment underwent morphological change on LNCaP. Treatment with 10, 50 and 100 μM BPA altered LNCaP cells morphology from cobblestone-like to a rounded shaped in a dose-dependent manner compared with control experiments where we found no differences in cellular size and/or morphology (Figure 1A). We next assessed proliferation and viability upon BPA treatment by fluorescence-activated cell sorting (FACS) analysis and MTT assay. BPA treatment has a slight inhibitory effect at 10 μM whereas at 50 and 100 μM results in a large fraction of cells which remain arrested in G0/G1, compared with the control cells showing an increased fractions in S and G2/M phases in a time dependent manner (Figure 1B). Cell viability was also determined by a MTT assay, which reveals that in cycling LNCaP cells the growth from 24 to 72 hrs is substantially blocked by BPA treatment, so that the difference of viable cell number, which is almost negligible after 24 hrs of treatment, strongly increases after 48 and even more after 72 hrs (Figure 2A). Interestingly, we found no increased LNCaP cells death in response to BPA treatment even at 100 μM concentration. These data suggest that a decreased LNCaP cell growth rate observed upon BPA treatment may involve cycle arrest rather than an enhanced cell death (Figure 1B-2A). In fact, already after 24 hours a clear decrease of cells entering S-phase is observed (Figure 2A). These observations led us to assess whether BPA treatment affects LNCaP to form colonies. We then performed a colony formation assay (Figure 2B). Consistent with FACS analysis, BPA treatment results in a full inhibition of colony number compared to control experiments (Figure 2B).

Taken together, these data clearly show that BPA induced cell cycle arrest and inhibition of colony formation in LNCaP cells.

### BPA abolishes the expression of cyclin D1 and induces expression of cell cycle inhibitors p21 and p27

Having established that 50 and 100 μM BPA inhibit cell cycle progression in human prostate cancer LNCaP cells, we next investigated the molecular mechanism by which BPA induced cell cycle arrest. First, we analyzed the expression of key regulatory proteins of cell cycle. We performed immunoblot analysis of cyclin D1, which is a key target in response to proliferative signals leading in mammalian cells to its expression level during the cell cycle in G1 phase [18, 19]. Immunoblotting results were consistent with FACS analysis (Figure 1B), showing a sustained expression level of cyclin D1 with 10 μM BPA treatment compared to the control cells whereas 50 and 100 μM BPA markedly decrease cyclin D1 expression after 48 and 72 hours. It is worth noting that high BPA concentration (100 μM) completely abolishes cyclin D1 expression even after 24 hours of treatment (Figure 2C). Next, we analyzed the expression of cell cycle inhibitor proteins. Decreased expression of cyclin D1 at 50 and 100 μM BPA leads to strongly increased expression of a well known cell cycle protein inhibitor p21 (Figure 2C). Interesting, 10 μM BPA slightly increases p21 cell cycle inhibitor level after 72 hrs, whereas treatment with 50 and 100 μM BPA strongly increases p21 expression even at 24 hrs (Figure 2C). The observed increase in p21 protein level after BPA treatment was even more clear-cut after 72 hours [20] (Figure 2C). ERK2 expression was used as a loading control during the time course (Figure 2C, lower panel). Accumulating evidence suggests that loss of p27 protein is a potential negative prognostic factor in human breast cancer [21] and in human prostate adenocarcinoma [22]. Therefore, we analyzed the p27 expression, which is a cell cycle regulator involved in the cell cycle G1 phase arrest, in response to BPA [23]. Consistent with p21 regulation upon BPA treatment, p27 expression is significantly increased following 50 μM BPA and even more clear-cut at 100 μM. Of note, 100 μM BPA induced markedly p27 expression even after 24 hours. In addition, Figure 2C showed that, at lower concentration, 10 μM BPA induces a slight increase in p27 expression after 24 hours, while we observed a significantly p27 increase after 48 and 72 hours (Figure 2C). These findings suggest that BPA induces cell cycle arrest in LNCaP cells by cyclin D1 and p21/p27 decreased or increased expression, respectively.

### BPA stimulates ERK but not Akt/PKB activity

**Figure 3:**
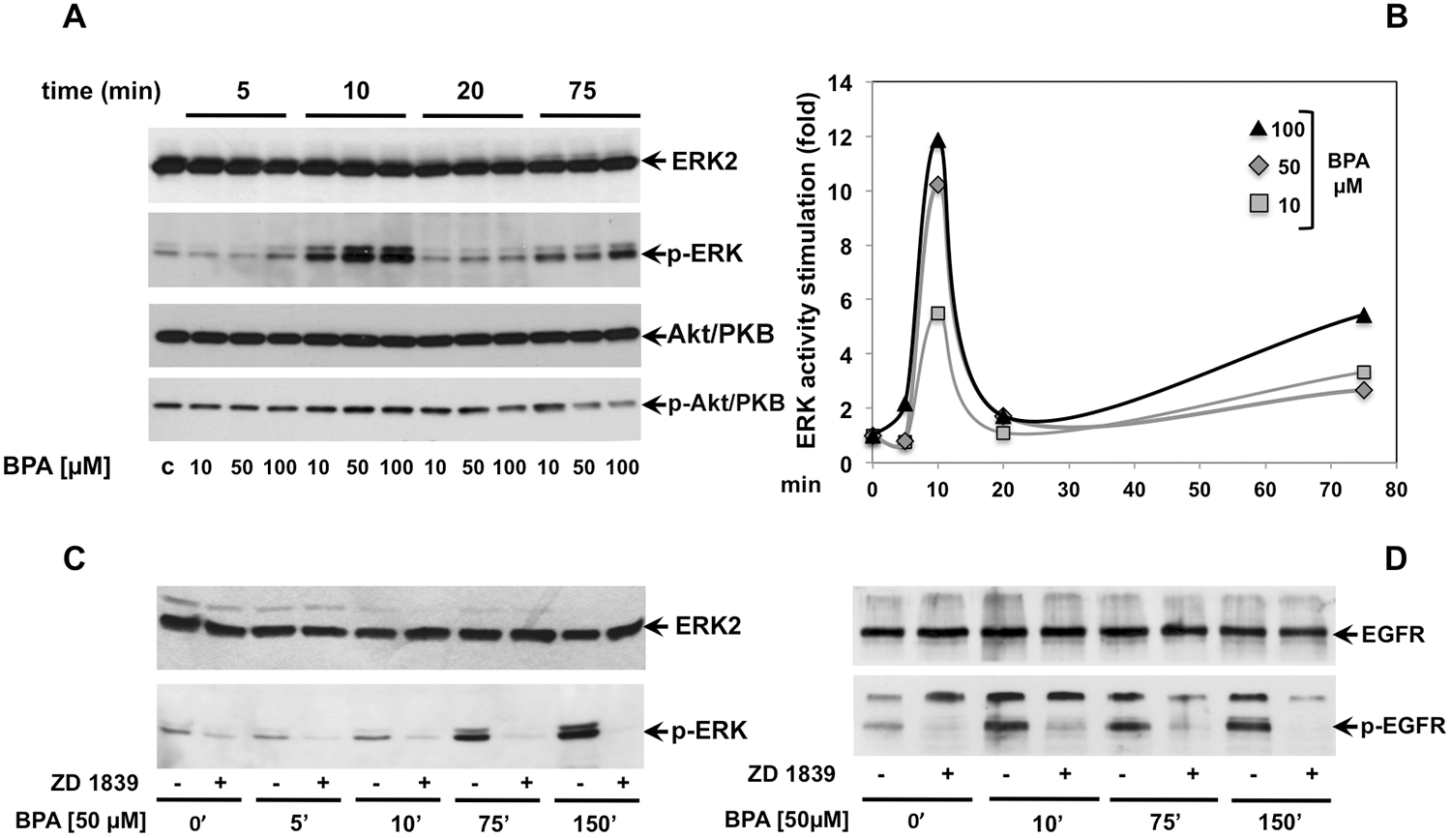
BPA stimulates ERK, but not Akt/PKB phosphorylation through epidermal growth factor receptor (EGFR) In **(A)**, LNCaP cells were left untreated or treated for the indicated times with Bisphenol A (BPA at 10, 50 and 100 μM). p-ERK activation (p-ERK) was analyzed in lysate proteins using the appropriate antibody. Filters were re-probed with the anti-ERK2 antibody. Akt/PKB activation (p-Akt/PKB) was analyzed using the anti p-Ser473 Akt/PKB Ab. Filters were reprobed with anti-Akt/PKB (Akt/PKB) antibody. The graph in **(B)** represents the level of p-ERK normalized to ERK2 from two independent experiments. In **(C** and **D)**, LNCaP cells were left untreated or treated for the indicated times with Bisphenol A (BPA at 10, 50 and 100 μM) in presence or absence of 5 μM ZD 1839. ERK activation (p-ERK) and ERK2 were analyzed in lysate proteins using the appropriate antibodies. p-EGFR (Tyr 1068; p-EGFR) was analyzed in lysate proteins using the appropriate antibody. Filters were stripped and re-probed with the anti-EGFR antibody.

To investigate the signal pathway underlying the inhibition of cyclin D1 expression and stimulation of expression levels of p21 and p27 leading to cell cycle arrest upon BPA treatment, we screened the activation of ERK and Akt/PKB proteins, two known signaling transduction kinases required for proliferation and survival [24]. Immunoblot analysis showed that BPA treatment induces ERK activation in LNCaP cells, as seen by an increased level of phospho-ERK (Figure 3A–3B), with a striking peak after 10 minutes of treatment. Interesting, the kinetic of ERK activation revealed two peaks of ERK phosphorylation during the time course of BPA treatment. In fact, 10 μM BPA rapidly increased the ERK phosphorylation (about 5.5-fold increase on 10 min of treatment as compared to control cells) and a clear highly ERK phosphorylation when the cells were treated with 50 and 100 μM BPA (10.2-fold and 11.8-fold increase compared to control cells, respectively, Figure 3A-3B). Then, the ERK phosphorylation returns to baseline after 20 minutes towards an increase in ERK phosphorylation upon 75 min of treatment with a significant induction of ERK phosphorylation upon 100 μM BPA treatment (Figure 3A-3B).

We next investigated Akt/PKB activation, a downstream substrate of phosphatidyl-inositol 3 kinase (PI3-K) mainly involved in cell survival [25]. Consistent with FACS analysis and MTT assay (Figure 1B; Figure 2), BPA does not induce Akt/PKB phosphorylation, used as a readout of Akt/PKB activation, during the time course observed and at all the different BPA concentrations used. However, a slight reduction of Akt/PKB phosphorylation can be observed in cells treated for 75 minutes with 50 and 100 μM BPA. (Figure 3A)

These data suggested that BPA induces a specific signal transduction activation involved in the regulation of the proliferation pathway with no significant influence of apoptosis and survival pathways.

### ERK activation is mediated by epidermal growth factor receptor (EGFR)

**Figure 4:**
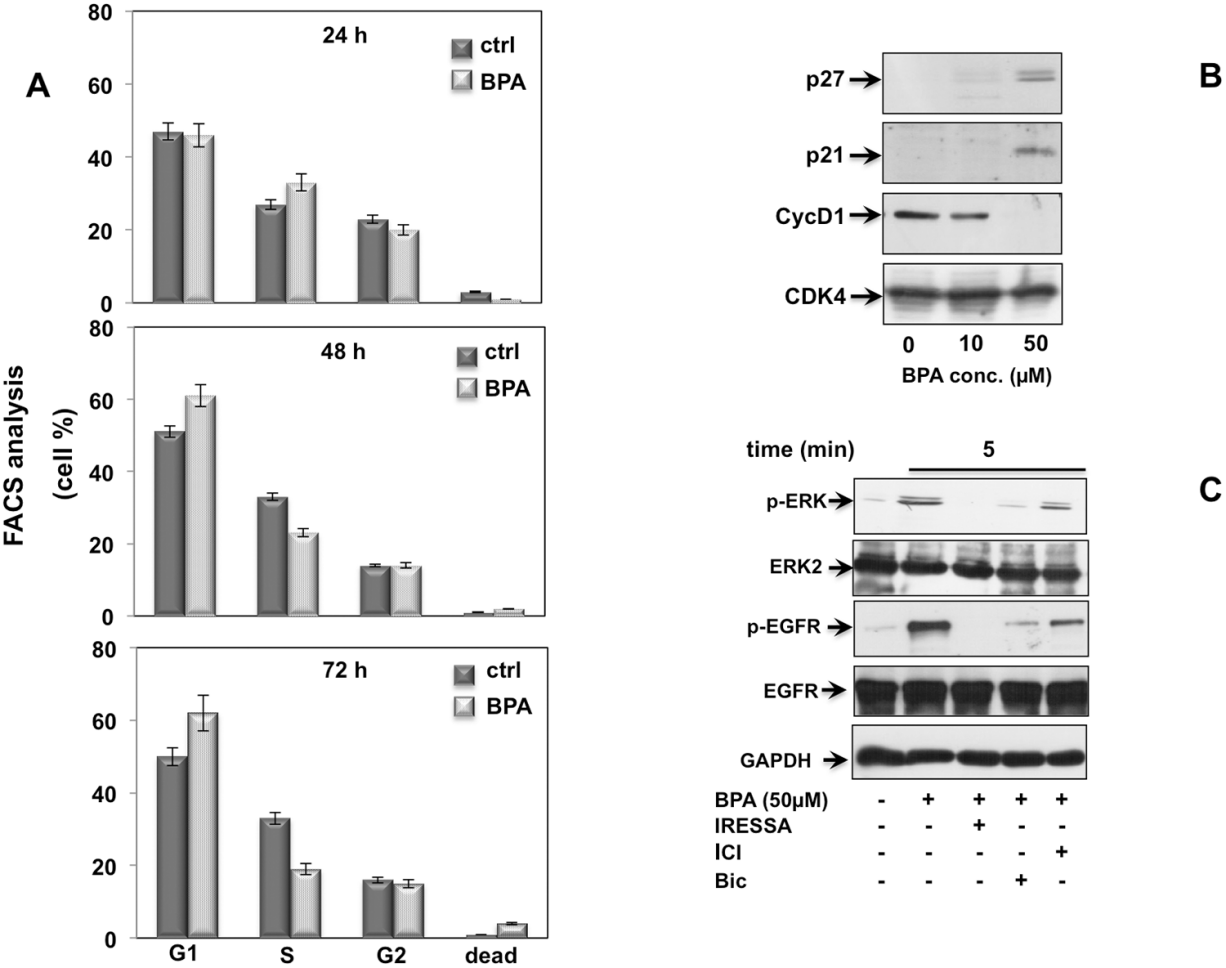
BPA induces cell cycle arrest in EPN cells In **(A)**, cycling EPN cells were left unstimulated (0) or stimulated for the indicated times with 50 μM Bisphenol A (BPA), Cells were re-suspended and analyzed by FACS, as described in Methods. The graphs show means from three independent experiments and the distinct cell cycle phases as reported in the graph legend. In **(B)**, EPN cells were untreated or treated with 10 or 50 μM Bisphenol A (BPA) for the indicated times and lysate proteins were analyzed by western blot, using antibodies against the indicated proteins (cyc D1: cyclin D1). In **(C)**, EPN cells were stimulated or unstimulated for 5 minutes with 50 μM Bisphenol A (BPA) in absence or presence of 5 μM ZD 1839 (ZD), 10 μM Bicalutamide (Bic) and 10 μM ICI 182,780 (ICI). Lysate proteins were immuno-blotted with the indicated antibodies.

Growth factors and steroid hormones activate ERK signaling pathway through high affinity receptors at membrane such as EGF, PDGF, insulin or intracellular receptors (estradiol, androgens, progestins). Previous studies showed that BPA induces a cross talk of membrane receptors with steroid receptors like androgen and estrogen receptors (AR and ER) and GPR30 (G protein-coupled estrogen receptor) leading to signal transduction pathway activation [26–28]. In addition, BPA regulates MCF-7 proliferation through STAT3 expression independent of EGFR activation [29]. We then investigated whether EGFR is required upstream of BPA-induced ERK activation in LNCaP cells. As shown in Figure 3C, BPA-induced EGFR activation results in increased level of EGFR phosphorylation within 5 min and sustained phosphorylation for all the time course examined (Figure 3C). BPA leads to specific EGFR activation, since use of EGFR-selective inhibitor ZD 1839 (Iressa) completely abolishes BPA-induced EGFR activation (Figure 3C). Consistent with this finding, BPA-induced EGFR phosphorylation correlates with the ERK activation following BPA treatment. Interesting, the BPA-induced ERK phosphorylation was completely blocked by the pre-treatment with the ZD 1839 inhibitor (Figure 3D). To confirm the relevance of BPA-induced cell cycle arrest in a more physiological context, we next investigated the effect of BPA in human non-transformed and differentiated prostate primary epithelial cell line EPN [30]. BPA treatment induces G2/M cell cycle arrest in EPN cells at all times analyzed (Figure 4A). To dissect the molecular mechanisms leading to cell cycle arrest, we next analyzed the cyclin D1 and the inhibitor p21, p27 expression. Western blot analysis shows that BPA induced p27 and 21 expression, whereas cyclin D1 was down regulated (Figure 4B). In addition, BPA treatment induced the phosphorylation of EGFR and ERK protein in EPN cells. Interestingly, EGFR and ERK phosphorylation was inhibited by the anti-estrogen ICI 182,780 or anti-androgen bicalutamide and by pretreatment with EGFR-selective inhibitor ZD 1839 (Iressa), (Figure 4C). These findings suggest that BPA effects observed in LNCaP cells clearly correlated with that shown in EPN cells, indicating a shared regulation of proliferation mechanisms in primary as well as in prostate cancer cells.

Taken together, these data indicated that BPA-induced EGFR activation occurs upstream of ERK leading to EGFR/ERK signal transduction activation and cell cycle arrest in LNCaP and non-transformed prostate cell line EPN.

### BPA stimulates p53 phosphorylation by a pathway involving ERK, EGFR, AR and ERβ

**Figure 5:**
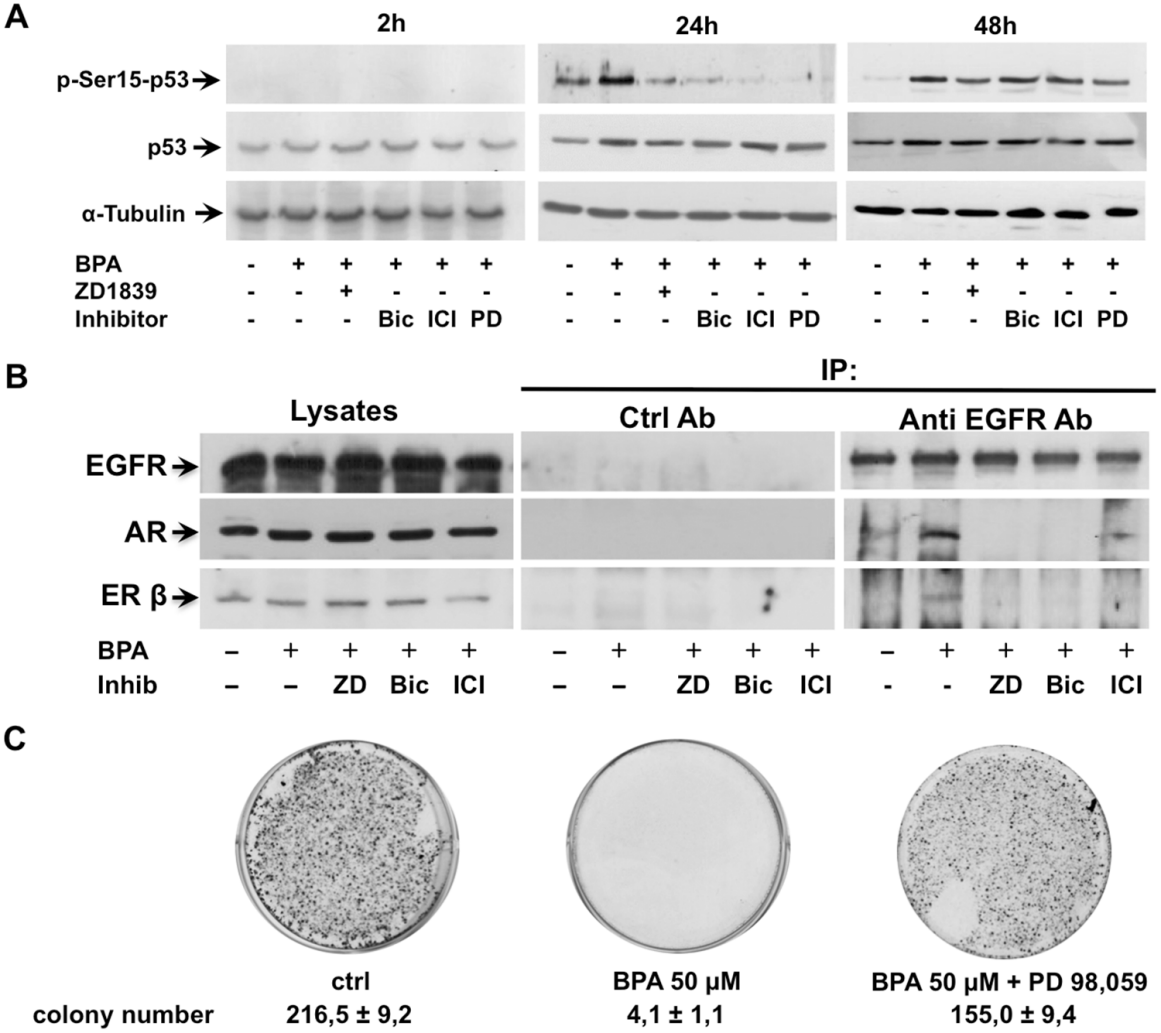
BPA stimulates p53 phosphorylation on residue Ser15 trough ERK, EGFR, AR and ERβ In **(A)**, LNCaP cells were left untreated or treated for the indicated times with 50 μM Bisphenol A (BPA) in absence or presence of 10 μM Bicalutamide (Bic), 10 μM ICI 182,780 (ICI) and 20 μM PD 98,059 (PD). Phosphorylation of p53 on residue Ser15 (p-Ser15-p53) was analyzed in lysate proteins using the appropriate antibody. Filters were stripped and re-probed with the anti-p53 antibody and α-Tubulin antibody, as loading control. In **(B)**, LNCaP cells were stimulated or unstimulated for 5 minutes with 50 μM Bisphenol A (BPA) in absence or presence of 5 μM ZD 1839 (ZD), 10 μM Bicalutamide (Bic) and 10 μM ICI 182,780 (ICI). Lysate proteins were immune-precipitated with EGFR antibody or non specific mouse immunoglobulin (Ctrl Ab) as control. Proteins in immune complexes were detected by Western blot using appropriate antibodies against indicated proteins. In **(C)**, ERK inhibition rescues the BPA-treated LNCaP cell ability to form colonies. LNCaP cells were seed in 6 well plates and stimulated or unstimulated with 50 μM Bisphenol A (BPA) in absence or presence of 20 μM PD 98,059. After 12 days the cells were fixed, stained and counted as reported in Materials and Methods.

As p53 is required to cell cycle arrest and survival under different conditions, we investigated whether p53 promotes inhibition of cellular proliferation in LNCaP cells upon BPA treatment. It was reported that activation of G protein-coupled receptor 30 (GPR30) induces cells cycle arrest in estrogen receptor negative (ER-) breast cancer cells through EGFR/ERK signal transduction pathway. The activation of EGFR/ERK pathway-mediated cell cycle arrest leads to increased expression of p53 and its phosphorylation on residue Ser15, which is crucial for its nuclear translocation and inhibition of p53 ubiquitination and degradation [31]. In addition, GPR30 has been shown to inhibit cell proliferation of androgen-independent PC3 prostate cancer cells by Erk1/2, c-jun/c-fos mediated p21 upregulation [32]. Consistent with these findings, we observed that 50 μM BPA induces a significant p53 Ser15 phosphorylation after 24 hours BPA treatment, which is inhibited by the EGFR inhibitor ZD 1839 and ERK inhibitor PD 98,059 (Figure 5A). Interestingly, BPA-mediated p53 Ser15 phosphorylation is also completely abolished by the AR antagonist bicalutamide and ER antagonist ICI 182,780 (Fulvestrant) (Figure 5A). Of note, the 48 hrs BPA-induced p53 Ser15 phosphorylation was independent on the EGFR and ERK inhibitors as well as steroid receptor antagonists. Having shown that BPA inhibits the LNCaP colony formation (Figure 2B), we next investigated whether this effect was reverted by pre-treatment with the ERK inhibitor PD 98,059. Our data show that treatment with PD 98,059 rescued the ability of LNCaP cells to form colonies despite the presence of BPA (Figure 5C).

These findings suggested that BPA on the early phase induced cell cycle arrest mediated by the activation of the EGFR/ERK signal pathway with the engage of steroid receptors leading to p53 phosphorylation, then, in the later phase, p53 activation results independent on the engagement of EGFR/ERK/steroid receptors.

### EGFR activation induced a protein-protein interaction between EGFR, AR and ERβ

Having shown that BPA induced cell cycle arrest through a cross-talk between EGFR and steroid receptors (AR and ERβ), we next assessed whether BPA promotes an interaction between EGFR and steroid receptors. To investigate whether BPA promotes an interaction between EGFR and steroid receptors LNCaP cells were treated in preliminary experiments with 50 μM BPA for times ranging from 5 to 120 minutes and cell lysates immunoprecipitated with anti EGFR antibodies (not shown). When immunoprecipitates were probed with anti-EGFR, anti-ERβ and anti-AR antibodies, Western blots show a clear, rapid and transient co immuno-precipitation of EGFR, AR and ERβ after 5 minutes of BPA treatment, with a much stronger association of AR than ERβ with EGFR. Figure 5B shows that BPA triggered a transient EGFR interaction with AR and ERβ within 5 min. and, interestingly, the steroid receptor antagonists disrupt the EGFR/AR/ERβ interaction. Furthermore, this interaction is also inhibited by EGFR inhibitor, suggesting that EGFR activation/phosphorylation is required for the cross-talk between EGFR, AR and ERβ.

## DISCUSSION

In this manuscript we have investigated the effect of Bisphenol A in human prostate cancer LNCaP cells and in human non-transformed epithelial prostate cells EPN. It was reported that 1 nM BPA treatment induces cell proliferation in human prostate cancer cells LNCaP which contain an androgen receptor (AR) point mutation (AR-T877A) frequently associated in patients with advanced prostate cancers refractory to hormone therapy [29]. This effect on cell proliferation is mediated by AR transcriptional activity. Consistent with this finding, BPA action seems related to the presence of the AR-T877A mutated variant leading the BPA to act as an agonist [33]. Previous study has shown that at higher doses (e.g. 10 μM) BPA causes growth inhibition of androgen-dependent prostate cancer cell lines (LNCaP and LAPC-4) [34]. However, the growth inhibitory effect at higher doses was observed only in androgen-dependent prostate cancer cells and mechanism underlying this inhibition is elusive.

Using the prostate cancer LNCaP cell line, which is also endowed with AR-T877A mutated variant, we observe that 50 and 100 μM BPA causes cell cycle arrest which is mediated by a non-transcriptional mechanism involving EGFR, ERK, AR, and ERβ. On the basis of the findings described in this manuscript, we propose that BPA binds to AR and ERβ inducing their association with EGFR. Physical and/or functional interaction between EGFR and steroid receptors have been already described [7, 35, 36]. EGFR activation is a hallmark of cross talk between growth factors and steroid receptors, which regulates cell growth, through the RAS/MEK/ERK signaling cascade. Interestingly, the BPA-induced inhibition of cell cycle and the EGFR/ERK pathway activation were also observed in normal prostate EPN cells, which are endowed with a wild-type (WT) AR receptor, indicating that BPA action in prostate cells is not restricted to a specific cancer cell line or linked to a specific AR mutated variants. Accumulating evidence indicates that GPR30, a steroid-binding affinity receptor, mediates cell cycle regulation in endocrine-related cancer [26, 31, 32, 37]. BPA is reported to activate GPR30/EGFR/ERK transduction pathway in SKBR3 breast cancer cells and cancer-associated fibroblasts (CAFs) lacking the classical estrogen receptor (ER) [26]. Of note, G-1, a selective GPR30 agonist, causes cell cycle arrest either in a GPER-dependent manner or in a GPER-independent manner [32, 37]. GPR30/ERK signaling activation leads to block of proliferation in androgen-independent PC3 prostate cancer cells [32], whereas G-1 agonist causes cell cycle arrest in HEK-293 and MDA-MB231 breast cancer GPER-negative cells and in KGN ovarian cancer cells siRNA knockdown of GPER [37]. Based on this evidence, GPR30 may be involved in the BPA action through either synergizing the classical steroid receptor action or replacing them, providing an alternative signaling in cells that do not express both AR and ER.

**Figure 6:**
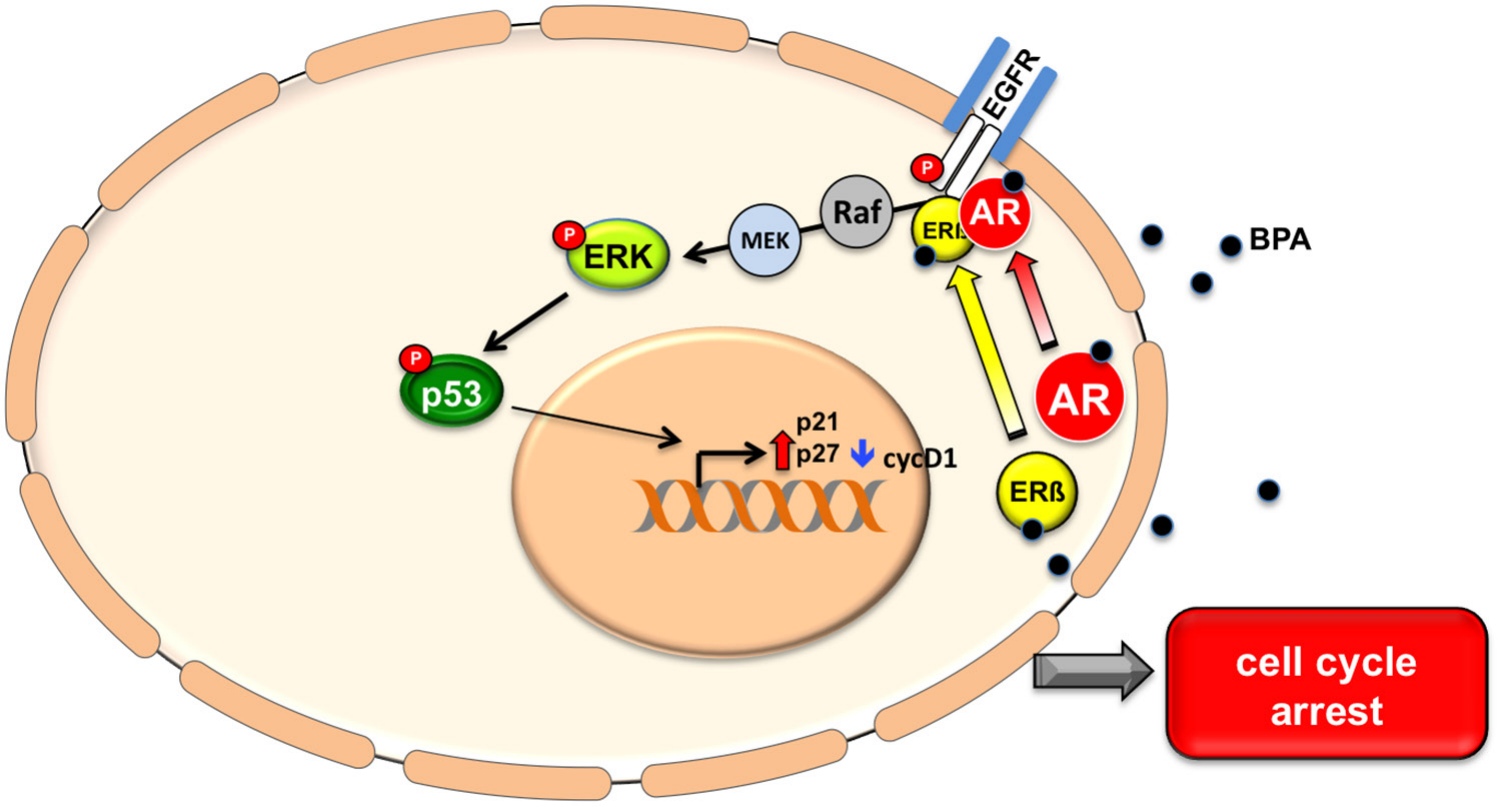
A model of BPA activation pathway Bisphenol A stimulates EGFR phosphorylation and triggers EGFR/AR/ERβ complex assembly. BPA treatment induces ERK activity and the consequent phosphorylation of p53 on residue Ser15 and hence p53 stabilization. This results in increased p27 and p21 expression levels and cyclin D1 protein down regulation, which lead to cell cycle arrest.

ERK activation is generally related to stimulation of cell cycle progression and growth stimulation. Nevertheless, it has recently demonstrated that other substances such as Sophoridine can induce S-phase cell cycle arrest through the ERK signaling [38]. We observed that in LNCaP cells ERK activation induces p53 phosphorylation on residue Ser15, which promotes its nuclear translocation and ubiquitination inhibition leading to enhanced p21 transcription. This observation indicates that p53 may be responsible for the cell cycle arrest observed upon BPA treatment (Figure 6).

These findings whereas provide new information about the BPA action on human cells, also raise some questions, first of all, about the possible significance “*in vivo*” of the effects of the exposure to high BPA concentrations. At this regard, it can be argued that is rather difficult that such concentrations can be reached in living cells or biological fluids. Nevertheless, it cannot ruled out that due to widespread presence of this substance as a contaminant in food samples, including vegetables and meat, its long half-life and ability to bind to several cell proteins, very high concentrations can occur under some circumstances in specific micro-environments. Another question to be addressed concerns the pathophysiological impact of BPA that these findings suggest. It has recently shown that neonatal bisphenol A exposure induces meiotic arrest and apoptosis of spermatogenic cells in male mice [39] and that trans-generational exposure to BPA promotes prostate developmental alterations. It has been observed that high BPA concentration promotes antiproliferative effects during neonatal prostate development in male and female gerbils [40, 41]. BPA also causes erectile dysfunction, reduction in the smooth muscle content with an increase in fat deposition in the penile corpora cavernosa of male rats [42]. These effects are consistent with the BPA inhibitory effect on cell cycle progression described here.

The relationship between cell cycle arrest induced by BPA and prostate cancer deserves careful consideration. “*In vitro*” assays revealed that BPA alters 5-alpha-dihydrotestosterone binding to AR-T877A likely through noncompetitive inhibition [13]. Consistent with the findings described here, at high BPA concentrations, LNCaP prostate cancer cell and EPN cell proliferation rate is decreased. This evidence may suggest that BPA or BPA derivatives antagonize androgen action and could be used to regulate the cell growth of prostate cancers expressing steroid receptors, independently on their apparent hormone responsiveness. However, further work is needed to validate this hypothesis.

Our study provides new insight into a mechanism by which BPA could drive prostate cancer cells toward hormone refractoriness. It suggests that BPA induces hormone resistance not only by its receptor antagonist properties but, more likely, by arresting cycle progression of hormone depending cells by a specific signaling pathway and providing a growth advantage for the cells lacking steroidal hormone or where AR and ERβ signaling is impaired.

In conclusion, the data of this manuscript indicate that high BPA concentrations inhibit AR dependent prostate cell growth. This mechanism could be responsible for impaired prostate development but also contribute to prostate cancer progression toward a more aggressive hormone refractory phenotype.

## MATERIALS AND METHODS

### Antibodies and reagents

Antibodies for biochemical studies were as follows from Cell Signaling Technology: anti-p-EGFR Tyr 1068 (catalog 2234), anti-p-Akt/PKB (catalog 9271), anti-Akt/PKB (total) (catalog 9272). Anti-p-ERK1/2 Tyr 204 (catalog sc7383), anti-ERK2 (total) (catalog sc154), anti-p53 (catalog sc126), anti-p21 (catalog sc397), anti-p27 (catalog sc528), anti-EGFR antibody for IP (A-10, catalog sc373746), anti-AR (catalog N20) was from Santa Cruz Biotechnology. Anti-cyclin D1 (catalog 33-3500) was from Invitrogen. Anti-EGFR for WB (catalog 06-847) was from Millipore. Anti-p-Ser15-p53 (catalog MAB1839) was from R&D Systems. Anti-ERβ (catalog 06-629) was from Upstate. Anti-tubulin was from Sigma-Aldrich. The tyrosine kinase inhibitor of the epidermal growth factor receptor (EGFR), ZD 1839, was from Selleckchem. Bisphenol A (BPA) was from Sigma-Aldrich. The antiandrogen bicalutamide (Casodex) was from Sigma-Aldrich. The antiestrogen ICI 182,780 was from Astra-Zeneca. The MEK-1 inhibitor PD 98,059 was from Sigma-Aldrich. All other reagents were of chemical grade.

### Cells

Human Prostate cancer cells LNCaP were from ATCC and cultured as reported [43, 44]. Human non-transformed epithelial prostate cells EPN [30] were cultured in DMEM modified Eagle’s medium supplemented with 7% fetal bovine serum with antibiotics.

### Contrast phase microscopy

Cells were left untreated or treated with 10, 50 or 100 μM Bisphenol A for the indicated times. Fields were analyzed with DMIRB inverted microscope (Leica) using N-Plan 10x objective (Leica). Images were captured using a DFC 450C camera (Leica) and acquired with Application Suite (Leica) software. They are representative of at least three independent experiments, each performed in duplicate.

### Fluorescence-activated cell sorting (FACS) analysis

For fluorescence-activated cell sorting (FACS) analysis, LNCaP cells (2.0 × 10^5^ for each experimental point) were left untreated or treated for the indicated times with Bisphenol A (10, 50 or 100 μM). Cells were collected, resuspended in 400 μl of a buffer containing 0.1% NP-40, 0.1% sodium citrate, 1 mg/ml RNAse A, 50 μg/ml propidium iodide and 0.1 mM EDTA. Cells were then incubated in the dark for 1h and samples were analyzed by a fluorescence-activated cell sorting (FACS) Calibur flow cytometer using Cell Quest software (Becton Dickinson, BD Biosciences). Results from three independent experiments were analyzed using Cell Quest software (Becton Dickinson) and ModFit LT version 3 Software (Verity, Topsham, ME, USA).

### Signaling studies

Total lysates after the indicated stimulation were prepared essentially as described [45]. The proteins were resolved by 10% sodium dodecyl sulfate–polyacrylamide gel electrophoresis (SDS-PAGE), transferred to polyvinylidene difluoride membranes (Immobilon-P; Millipore, Bedford, MA) and probed with the indicated antibodies, followed by detection of immunoreactive proteins by an enhanced chemiluminescence system (PIERCE^TM^ ECL from Thermo Fisher Scientific). Proteins from 2mg of total lysates were immunoprecipitated using the indicated antibodies as described [43]. Mouse immunoglobulins were used as control (Jackson Immunoresearch) and Western blot analysis was performed essentially as described [45]. The ECL system (PIERCE^TM^ ECL from Thermo Fisher Scientific) was used to reveal immune-reactive proteins.

### Cell viability and colony formation assay

Cell viability was determined using the colorimetric WST-1 (2-[4-Iodophenyl]-3-[4-nitrophenyl]-5-[2,4-disulfophenyl]-2H-tetrazolium, monosodium salt cell viability assay according to the manufacturer’s instructions (Roche Diagnostics, Mannheim, Germany) as previously reported [46]. Briefly, cells were plated in 96-well plates plates at a density of 1 × 10^4^ cells per well in corresponding growth media in triplicates. Cell lines were allowed to proliferate for 24 h to reach exponential growth rates. Cells were then incubated for 48–72 h at cell culture conditions and proliferation assays were performed according to the manufacturer’s protocol. Chemical reduction of the WST-1 dye was determined by optical density absorption analysis at 450 nm, using an ELISA plate reader (Tecan Group Ltd, Männedorf, Switzerland). The fluorescence intensity measured at excitation wavelength of 550 nm and emission at 590 nm. Colony formation assays were performed on cycling LNCaP cells plated in 10 cm plates. LNCaP cells were allowed to grow in appropriate cultured medium and treated or untreated with 50 μM BPA in presence and in absence of 20 μM PD 98,059. Fresh media were supplied every 3 days. After 12 days, the colonies were fixed with 3.5% formaldehyde/80% methanol and stained with 3-(4,5-dimethylthiazol-2-yl)- 2,5-diphenyltetrazolium bromide (Sigma, St Louis, MO, USA). The stained colonies were photographed and the number colonies with sizes ≥ 1mm were counted using the ImageJ software (National Institutes of Health, USA) and expressed as mean±S.E.M of triplicated. Each assay was performed in triplicated and repeated twice.

### Statistical analysis

Experiments were performed in triplicate, which yielded highly consistent results. All data are presented as mean±SEM unless otherwise stated. The Differences between values observed after the various treatments were analyzed using the Student’s t test for unpaired or paired observations. Results were considered significant at a value of p < 0.05. When shown data were analyzed using the NIH Image J program and expressed as relative increase. All authors have read and agreed to the manuscript as written.
