# Correction: Bisphenol A induces cell cycle arrest in primary and prostate cancer cells through EGFR/ERK/p53 signaling pathway activation

**DOI:** 10.18632/oncotarget.28845

**Published:** 2026-03-27

**Authors:** Antonio Bilancio, Paola Bontempo, Marzia Di Donato, Mariarosaria Conte, Pia Giovannelli, Lucia Altucci, Antimo Migliaccio, Gabriella Castoria

**Affiliations:** ^1^Department of Biochemistry, Biophysics and General Pathology, University of Campania “L. Vanvitelli”, Naples, Italy; ^2^IRCCS, SDN, Naples, Italy; ^*^These authors have contributed equally to this work

**This article has been corrected:** In Figure 1A, two micrograph images of LNCaP cells treated with 10 μM Bisphenol A for 48 and 72 hours overlap two images of LNCaP cells treated with 50 μM Bisphenol A for 24 and 48 hours. The authors provided unmodified images from the original experiments and declared that the images in Figure 1A are only illustrative of the results of the work and have not been used for quantization, thus these corrections do not change the results or conclusions of this paper. The corrected Figure 1A, produced using the original data, is shown below.

Original article: Oncotarget. 2017; 8:115620–115631. 115620-115631. https://doi.org/10.18632/oncotarget.23360

**Figure 1:**
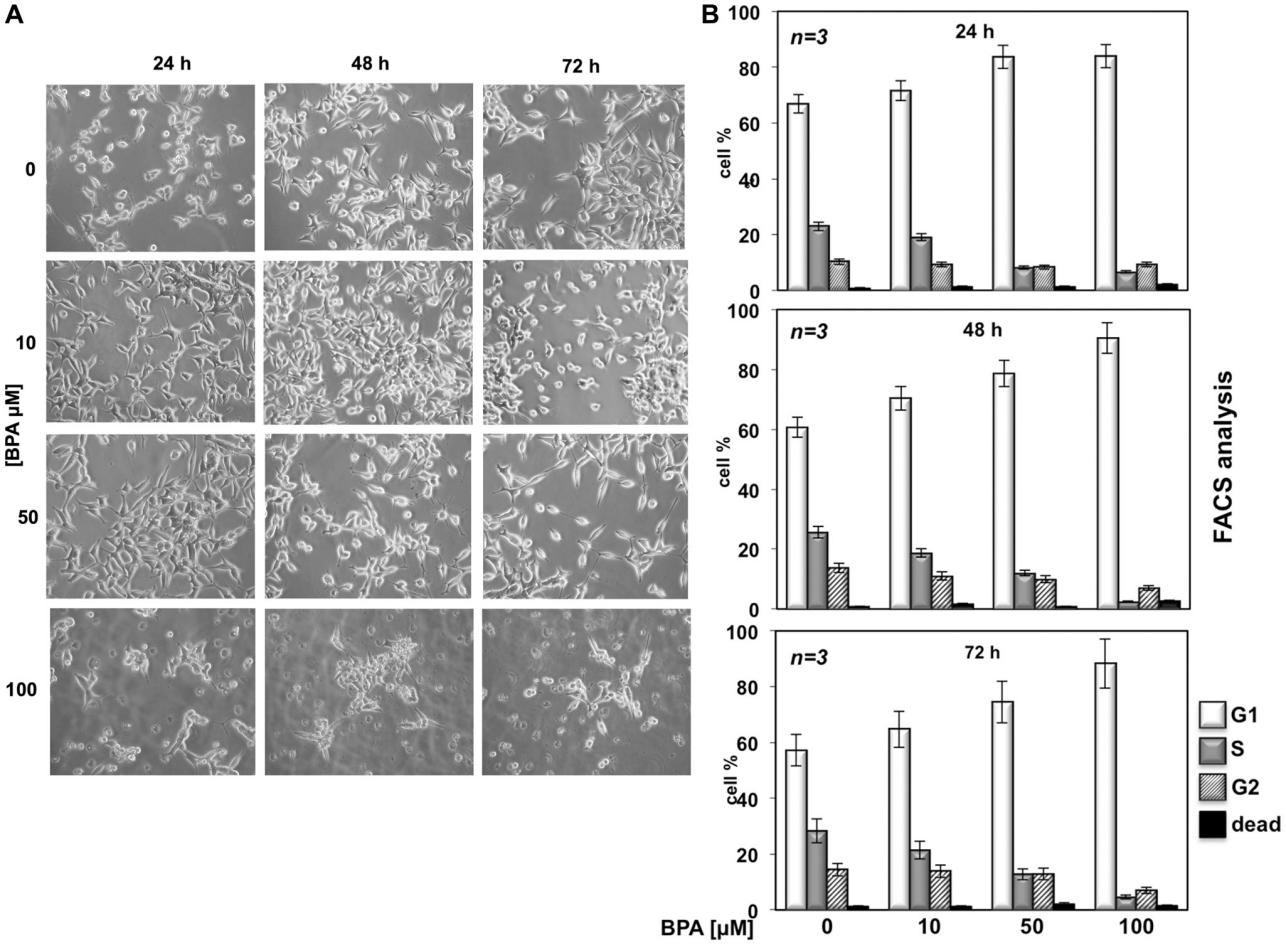
BPA affects LNCaP cell proliferation. In (**A**), LNCaP cells were plated at the same confluence in 100 mm dishes and unstimulated (0) or stimulated with 10, 50 or 100 μM Bisphenol A for 24, 48 and 72 hours. Images were captured with DMIRB inverted microscope (Leica) using N-Plan 10x objective (Leica) and a DFC 450C camera (Leica). They were analyzed with Application Suite (Leica) software and are representative of at least three independent experiments, each performed in duplicate. In (**B**), cycling LNCaP cells were left unstimulated (0) or stimulated for the indicated times with Bisphenol A (BPA at 10, 50 and 100 μM). Cells were re-suspended and analyzed by FACS, as described in Methods. The graphs show means from three independent experiments and the distinct cell cycle phases as reported in Methods.

